# 8-Bromo­naphthalen-1-amine

**DOI:** 10.1107/S1600536808012580

**Published:** 2008-05-03

**Authors:** Amy L. Fuller, Fergus R. Knight, Alexandra M. Z. Slawin, J. Derek Woollins

**Affiliations:** a Department of Chemistry, University of St Andrews, St Andrews KY16 9ST, Scotland

## Abstract

The title compound, C_10_H_8_BrN, was obtained by slow addition of sodium azide to 8-bromo-1-naphthoic acid, followed by addition of aqueous ammonia. The crude product was crystallized from petroleum ether to give pink crystals. Compared to other 1,8-disubstituted naphthalene compounds, this compound exhibits less strain between the 1 and 8 substituents. Additionally, the NH protons form both intra- and inter­molecular hydrogen bonds. The naphthalene units are arranged in a herring-bone stacking motif.

## Related literature

For examples of sterically crowded 1,8 dichalcogen naphthalenes, see: Aucott *et al.* (2004 ▶). For the synthesis, see: Herbert *et al.* (1987 ▶).
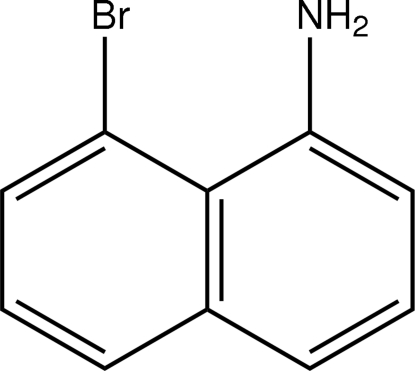


## Experimental

### 

#### Crystal data

C_10_H_8_BrN
*M* *_r_* = 222.08
Monoclinic, 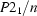

*a* = 13.6692 (14) Å
*b* = 4.1579 (4) Å
*c* = 15.8256 (16) Å
β = 109.941 (3)°
*V* = 845.52 (15) Å^3^
*Z* = 4
Mo *K*α radiation
μ = 4.81 mm^−1^
*T* = 125.1 K
0.35 × 0.13 × 0.09 mm

#### Data collection

Rigaku SCXmini diffractometer
Absorption correction: multi-scan (*ABSCOR*; Higashi, 1995 ▶) *T*_min_ = 0.381, *T*_max_ = 0.649
6823 measured reflections
1527 independent reflections
1281 reflections with *F* ^2^ > 2σ(*F* ^2^)
*R*_int_ = 0.061

#### Refinement

*R*[*F* ^2^ > 2σ(*F* ^2^)] = 0.051
*wR*(*F* ^2^) = 0.116
*S* = 1.10
1527 reflections
110 parameters
H-atom parameters constrained
Δρ_max_ = 1.76 e Å^−3^
Δρ_min_ = −0.39 e Å^−3^

### 

Data collection: *SCXmini* (Rigaku/MSC, 2006 ▶); cell refinement: *PROCESS-AUTO* (Rigaku, 1998 ▶); data reduction: *PROCESS-AUTO*; program(s) used to solve structure: *SHELXS97* (Sheldrick, 2008 ▶); program(s) used to refine structure: *SHELXL97* (Sheldrick, 2008 ▶); molecular graphics: *CrystalStructure* (Rigaku/MSC, 2006 ▶); software used to prepare material for publication: *CrystalStructure*.

## Supplementary Material

Crystal structure: contains datablocks global, I. DOI: 10.1107/S1600536808012580/si2087sup1.cif

Structure factors: contains datablocks I. DOI: 10.1107/S1600536808012580/si2087Isup2.hkl

Additional supplementary materials:  crystallographic information; 3D view; checkCIF report

## Figures and Tables

**Table 1:** Hydrogen-bond geometry (Å, °)

*D*—H⋯*A*	*D*—H	H⋯*A*	*D*⋯*A*	*D*—H⋯*A*
N1—H1*a*⋯Br1	0.98	2.27	3.070 (3)	138
N1—H1*b*⋯N1^i^	0.98	2.20	3.073 (5)	148

Symmetry code: (i) 
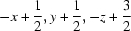
.

**Fig. 1.**
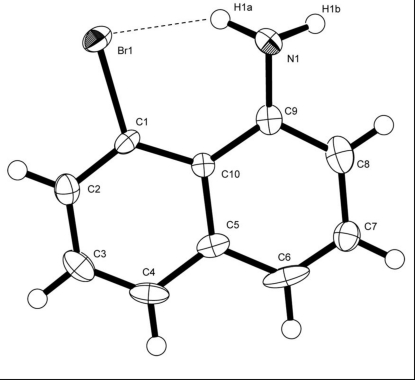
The structure of the title compound with labelled atoms and with displacement ellipsoids for non-H atoms drawn at the 50% probability level. The intramolecular hydrogen bond is indicated by a dashed line.

**Fig. 2.**
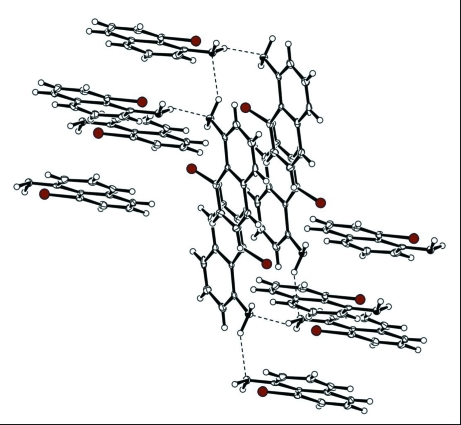
Packing diagram illustrating the intermolecular hydrogen bonding.

## supplementary crystallographic information

### Comment

Contrary to the formally bonded (and sterically strained) S—S or Te—Te atoms in positions 1 and 8 in previous naphthalene compounds (Aucott *et al.*, 2004), the title compound C_10_H_8_BrN exhibits a somewhat unstrained, intramolecular non-bonded Br1···N1 distance of 3.070 (3) Å. The molecular structure of the title compound is shown in Figure 1. An intramolecular hydrogen bonding interaction between the N1—H1a···Br1 enables a close nonbonding Br1···N1 distance and at the same time leaves the molecule relatively unstrained.

The other NH proton is involved in the intermolecular hydrogen bond (Table 1) N1—H1b···N1 and forms an infinite zigzag chain. These chains have normal hydrophobic contact to each other (Figure 2). The naphthalene units are arranged in a herringbone stacking motif.

### Experimental

The title compound was prepared by a method previously described (Herbert *et al.*, 1987). Sodium azide (3.10 g, 0.048 mol) was added over a 10 minute period to a stirred suspension of 8-bromo-1-naphthoic acid (2.0 g, 0.008 mol) in concentrated sulfuric acid (7 ml) and chloroform (7 ml) at 45° C. Each successive portion of sodium azide being added after the effervescence resulting from the previous addition had subsided. The mixture was stirred for 90 minutes at 45°C and added to water (140 ml), and it was made alkaline with aqueous ammonia and extracted with chloroform (3 *x* 140 ml). The combined extracts were dried with magnesium sulfate and evaporated to give the desired product. Yield 1.30 g (73%). The black crude product was recrystallized from 60–80 petroleum ether to give pink crystals.

### Refinement

C7 was refined subject to an ISOR constraint. All H atoms were included in calculated positions (C—H distances are 0.95 Å, N—H distances are 0.98 Å) and were refined as riding atoms with *U*_iso_(H) = 1.2 *U*_eq_(parent carbon atom) The N—H protons were refined subject to a distance constraint but with a riding thermal parameter.

Refinement of F^2^ against ALL reflections. The weighted *R*-factor *wR* and goodness of fit S are based on F^2^, conventional *R*-factors *R* are based on F, with F set to zero for negative F^2^. The threshold expression of F^2^ > 2sigma(F^2^) is used only for calculating *R*-factors(gt) *etc*. and is not relevant to the choice of reflections for refinement. *R*-factors based on F^2^ are statistically about twice as large as those based on F, and R– factors based on ALL data will be even larger.

### Crystal data

**Table:**

C_10_H_8_BrN	*F*_000_ = 440.00
*M**_r_* = 222.08	*D*_x_ = 1.744 Mg m^−^^3^
Monoclinic, *P*2_1_/*n*	Melting point: 359 K
Hall symbol: -P 2yn	Mo *K*α radiation λ = 0.71075 Å
*a* = 13.6692 (14) Å	Cell parameters from 7235 reflections
*b* = 4.1579 (4) Å	θ = 3.0–27.6º
*c* = 15.8256 (16) Å	µ = 4.81 mm^−^^1^
β = 109.941 (3)º	*T* = 125.1 K
*V* = 845.52 (15) Å^3^	Prism, pink
*Z* = 4	0.35 × 0.13 × 0.09 mm

### Data collection

**Table:**

Rigaku SCXmini diffractometer	1281 reflections with *F*^2^ > 2σ(*F*^2^)
Detector resolution: 6.85 pixels mm^-1^	*R*_int_ = 0.061
ω scans	θ_max_ = 25.4º
Absorption correction: multi-scan(ABSCOR; Higashi, 1995)	*h* = −16→16
*T*_min_ = 0.381, *T*_max_ = 0.649	*k* = −5→5
6823 measured reflections	*l* = −19→19
1527 independent reflections	

### Refinement

**Table:**

Refinement on *F*^2^	H-atom parameters constrained
Least-squares matrix: full	*w* = 1/[σ^2^(*F*_o_^2^) + (0.0516*P*)^2^ + 2.4354*P*] where *P* = (*F*_o_^2^ + 2*F*_c_^2^)/3
*R*[*F*^2^ > 2σ(*F*^2^)] = 0.051	(Δ/σ)_max_ = 0.002
*wR*(*F*^2^) = 0.116	Δρ_max_ = 1.76 e Å^−^^3^
*S* = 1.10	Δρ_min_ = −0.39 e Å^−^^3^
1527 reflections	Extinction correction: none
110 parameters	

### Special details

**Table:**

Geometry. All e.s.d.'s (except the e.s.d. in the dihedral angle between two l.s. planes) are estimated using the full covariance matrix. The cell e.s.d.'s are taken into account individually in the estimation of e.s.d.'s in distances, angles and torsion angles; correlations between e.s.d.'s in cell parameters are only used when they are defined by crystal symmetry. An approximate (isotropic) treatment of cell e.s.d.'s is used for estimating e.s.d.'s involving l.s. planes.

### Fractional atomic coordinates and isotropic or equivalent isotropic displacement parameters (Å^2^)

**Table:**

	*x*	*y*	*z*	*U*_iso_*/*U*_eq_	
Br(1)	0.31174 (4)	0.55948 (14)	0.53932 (4)	0.0293 (2)	
N(1)	0.3079 (3)	0.8778 (7)	0.71320 (17)	0.0237 (10)	
C(1)	0.4427 (3)	0.4617 (12)	0.6330 (3)	0.0198 (11)	
C(2)	0.5076 (4)	0.2842 (13)	0.6015 (3)	0.0257 (12)	
C(3)	0.6037 (4)	0.1842 (14)	0.6610 (4)	0.0304 (13)	
C(4)	0.6321 (4)	0.2554 (12)	0.7494 (4)	0.0274 (13)	
C(5)	0.5666 (4)	0.4409 (12)	0.7842 (3)	0.0224 (11)	
C(6)	0.5988 (4)	0.5102 (13)	0.8776 (4)	0.0349 (15)	
C(7)	0.5414 (4)	0.6701 (14)	0.9140 (3)	0.0263 (12)	
C(8)	0.4419 (4)	0.8017 (14)	0.8560 (3)	0.0314 (13)	
C(9)	0.4054 (3)	0.7493 (12)	0.7623 (3)	0.0206 (11)	
C(10)	0.4667 (3)	0.5569 (11)	0.7240 (3)	0.0157 (10)	
H(1a)	0.2955	0.8886	0.6486	0.020*	
H(2a)	0.4872	0.2298	0.5396	0.031*	
H(3a)	0.6495	0.0651	0.6393	0.037*	
H(4a)	0.6971	0.1804	0.7892	0.033*	
H(1b)	0.2991	1.0681	0.7464	0.057*	
H(6a)	0.6652	0.4367	0.9152	0.042*	
H(7a)	0.5638	0.7006	0.9772	0.032*	
H(8a)	0.4010	0.9253	0.8820	0.038*	

### Atomic displacement parameters (Å^2^)

**Table:**

	*U*^11^	*U*^22^	*U*^33^	*U*^12^	*U*^13^	*U*^23^
Br(1)	0.0265 (3)	0.0337 (3)	0.0210 (3)	0.0021 (2)	−0.0007 (2)	−0.0014 (2)
N(1)	0.023 (2)	0.020 (2)	0.029 (2)	0.0018 (19)	0.011 (2)	−0.0008 (18)
C(1)	0.018 (2)	0.025 (2)	0.014 (2)	−0.003 (2)	0.003 (2)	0.003 (2)
C(2)	0.034 (3)	0.021 (2)	0.025 (3)	−0.001 (2)	0.014 (2)	−0.001 (2)
C(3)	0.027 (3)	0.025 (3)	0.046 (3)	0.000 (2)	0.021 (2)	−0.001 (2)
C(4)	0.016 (2)	0.016 (3)	0.045 (3)	0.000 (2)	0.004 (2)	0.010 (2)
C(5)	0.020 (2)	0.016 (2)	0.026 (2)	−0.005 (2)	0.001 (2)	0.005 (2)
C(6)	0.021 (2)	0.022 (3)	0.043 (3)	−0.009 (2)	−0.013 (2)	0.016 (2)
C(7)	0.0278 (14)	0.0273 (15)	0.0247 (14)	−0.0040 (9)	0.0100 (9)	−0.0020 (9)
C(8)	0.035 (3)	0.026 (3)	0.038 (3)	−0.012 (2)	0.019 (2)	−0.010 (2)
C(9)	0.024 (2)	0.014 (3)	0.026 (2)	−0.009 (2)	0.011 (2)	−0.003 (2)
C(10)	0.015 (2)	0.014 (2)	0.019 (2)	−0.005 (2)	0.007 (2)	−0.0002 (19)

### Geometric parameters (Å, °)

**Table:**

Br(1)—C(1)	1.939 (4)	C(8)—C(9)	1.410 (7)
N(1)—C(9)	1.400 (5)	C(9)—C(10)	1.435 (8)
C(1)—C(2)	1.372 (8)	N(1)—H(1a)	0.98
C(1)—C(10)	1.420 (7)	N(1)—H(1b)	0.98
C(2)—C(3)	1.393 (7)	C(2)—H(2a)	0.95
C(3)—C(4)	1.350 (8)	C(3)—H(3a)	0.95
C(4)—C(5)	1.428 (8)	C(4)—H(4a)	0.95
C(5)—C(6)	1.420 (8)	C(6)—H(6a)	0.95
C(5)—C(10)	1.455 (6)	C(7)—H(7a)	0.95
C(6)—C(7)	1.304 (9)	C(8)—H(8a)	0.95
C(7)—C(8)	1.462 (7)		
			
Br(1)—C(1)—C(2)	112.2 (3)	C(5)—C(10)—C(9)	117.4 (4)
Br(1)—C(1)—C(10)	123.4 (4)	C(9)—N(1)—H(1a)	113.0
C(2)—C(1)—C(10)	124.4 (4)	C(9)—N(1)—H(1b)	106.2
C(1)—C(2)—C(3)	119.4 (5)	H(1a)—N(1)—H(1b)	120.9
C(2)—C(3)—C(4)	120.5 (6)	C(1)—C(2)—H(2a)	120.3
C(3)—C(4)—C(5)	121.4 (4)	C(3)—C(2)—H(2a)	120.3
C(4)—C(5)—C(6)	119.9 (4)	C(2)—C(3)—H(3a)	119.8
C(4)—C(5)—C(10)	119.9 (4)	C(4)—C(3)—H(3a)	119.8
C(6)—C(5)—C(10)	120.2 (5)	C(3)—C(4)—H(4a)	119.3
C(5)—C(6)—C(7)	123.0 (4)	C(5)—C(4)—H(4a)	119.3
C(6)—C(7)—C(8)	118.9 (5)	C(5)—C(6)—H(6a)	118.5
C(7)—C(8)—C(9)	121.5 (5)	C(7)—C(6)—H(6a)	118.5
N(1)—C(9)—C(8)	117.0 (5)	C(6)—C(7)—H(7a)	120.5
N(1)—C(9)—C(10)	124.1 (4)	C(8)—C(7)—H(7a)	120.5
C(8)—C(9)—C(10)	118.8 (4)	C(7)—C(8)—H(8a)	119.3
C(1)—C(10)—C(5)	114.4 (4)	C(9)—C(8)—H(8a)	119.3
C(1)—C(10)—C(9)	128.2 (4)		
			
Br(1)—C(1)—C(2)—C(3)	−177.8 (4)	C(4)—C(5)—C(10)—C(9)	−178.4 (5)
Br(1)—C(1)—C(10)—C(5)	176.3 (3)	C(6)—C(5)—C(10)—C(1)	−178.0 (5)
Br(1)—C(1)—C(10)—C(9)	−3.7 (8)	C(6)—C(5)—C(10)—C(9)	2.0 (7)
C(2)—C(1)—C(10)—C(5)	−2.0 (8)	C(10)—C(5)—C(6)—C(7)	1.7 (9)
C(2)—C(1)—C(10)—C(9)	178.0 (5)	C(5)—C(6)—C(7)—C(8)	−3.8 (9)
C(10)—C(1)—C(2)—C(3)	0.6 (9)	C(6)—C(7)—C(8)—C(9)	2.4 (9)
C(1)—C(2)—C(3)—C(4)	1.3 (9)	C(7)—C(8)—C(9)—N(1)	178.2 (5)
C(2)—C(3)—C(4)—C(5)	−1.6 (9)	C(7)—C(8)—C(9)—C(10)	1.2 (8)
C(3)—C(4)—C(5)—C(6)	179.7 (5)	N(1)—C(9)—C(10)—C(1)	0.0 (8)
C(3)—C(4)—C(5)—C(10)	0.1 (6)	N(1)—C(9)—C(10)—C(5)	−180.0 (4)
C(4)—C(5)—C(6)—C(7)	−177.9 (5)	C(8)—C(9)—C(10)—C(1)	176.8 (5)
C(4)—C(5)—C(10)—C(1)	1.6 (7)	C(8)—C(9)—C(10)—C(5)	−3.2 (7)

### Hydrogen-bond geometry (Å, °)

**Table:**

*D*—H···*A*	*D*—H	H···*A*	*D*···*A*	*D*—H···*A*
N1—H1a···Br1	0.98	2.27	3.070 (3)	138
N1—H1b···N1^i^	0.98	2.20	3.073 (5)	148

Symmetry codes:  (i) −*x*+1/2, *y*+1/2, −*z*+3/2.
